# Combining relation extraction with function detection for BEL statement extraction

**DOI:** 10.1093/database/bay133

**Published:** 2019-01-08

**Authors:** Suwen Liu, Wei Cheng, Longhua Qian, Guodong Zhou

**Affiliations:** School of Computer Science and Technology, Soochow University, Suzhou, China

## Abstract

The BioCreative-V community proposed a challenging task of automatic extraction of causal relation network in Biological Expression Language (BEL) from the biomedical literature. Previous studies on this task largely used models induced from other related tasks and then transformed intermediate structures to BEL statements, which left the given training corpus unexplored. To make full use of the BEL training corpus, in this work, we propose a deep learning-based approach to extract BEL statements. Specifically, we decompose the problem into two subtasks: entity relation extraction and entity function detection. First, two attention-based bidirectional long short-term memory networks models are used to extract entity relation and entity function, respectively. Then entity relation and their functions are combined into a BEL statement. In order to boost the overall performance, a strategy of threshold filtering is applied to improve the precision of identified entity functions. We evaluate our approach on the BioCreative-V Track 4 corpus with or without gold entities. The experimental results show that our method achieves the state-of-the-art performance with an overall F1-measure of 46.9% in stage 2 and 21.3% in stage 1, respectively.

## Introduction

Automatic extraction of biological network information is one of the most desired and challenging tasks in biomedical text mining research community. It needs to integrate biomedical knowledge buried in the literature into knowledge bases in a structured representation. Well-established standards to formally represent biological networks are the Systems Biology Markup Language (1), the Biological Pathway Exchange Language (2) and the Biological Expression Language (BEL) (http://www.openbel.org/) (3). Among them, BEL is gaining increasing attention for system biology applications because it combines the power of a formalized representation language with a relatively simple syntax designed to be both human readable and machine accessible.

Despite there have been multiple knowledge acquisition efforts in biomedical domain, such as Comparative Toxicogenomics Database (CTD) (4) and sbv IMPROVER initiative (5), a considerable amount of knowledge is still buried in the literature due to the high cost and special expertise needed for knowledge curation. For promoting the research on biomedical text mining technologies, BioCreative-V community proposed a challenging task of automatically extracting casual network information in BEL format from biomedical literature (http://www.biocreative.org/tasks/biocreative-v/track-4-bel-task/). BEL is designed to represent scientific findings in the field of life sciences in a structured form. BEL statements convey causal relationships (‘increases*’* and *‘*decreases*’*) between two BEL terms or among multiple BEL terms. BEL terms are formed using biomedical entities (gene/protein and chemical abundances, biological and pathological processes) together with functions that are used to modify entities [e.g. *deg()* (degradation)*, tloc()* (translocation)]. A concept of namespaces [e.g. Chemical Entities of Biological Interest (CHEBI)] and associated identifiers, e.g. a(CHEBI:‘nitric oxide’), is adopted to normalize entities in a flexible way.

Different from previous biomedical relation extraction (RE) tasks such as disease–gene association (6, 7) and protein–protein interaction (8, 9), where relationship is purely binary, the BEL tasks (BioCreative V Track 4 Task 1 and BioCreative VI Track 4 Task 2) aim to discover the hierarchical relations between biomedical entities, meaning that the relationship (‘increases’ or ‘decreases’) can hold among multiple entities and complex biomedical functions [such as *complex()* or *tloc()*] can also be involved. The goal of the BEL tasks is to extract the whole BEL statement from the sentence. It defines two evaluation stages depending on whether gold entities on the test set are given (stage 2) or not (stage 1). Taking as examples the following sentences and their corresponding BEL statements extracted from the BioCreative-V (BC-V) corpus (For easy reference, we mark the entity mentions in the sentences in italic type face.):

- We now demonstrate that *AKAP220* fragment is a competive inhibitor of *PP1c* activity (K(i) = 2.9 +/− 0.7 micrometer) (PMID: 11152471).
  p(HGNC:AKAP11) decreases act(p(HGNC:PPP1CC)).
- *UbcH7* is a ubiquitin-conjugating enzyme mediating *c-fos* degradation, transcription factor NF-kappaB maturation, human papilloma viru-mediated *p53* and *Myc* protein degradation, in vitro. (PMID: 10760570).
  cat(p(HGNC:UBE2L3)) increases deg(p(HGNC:FOS)).
  cat(p(HGNC:UBE2L3)) increases deg(p(HGNC:*MYC*)).
  cat(p(HGNC:UBE2L3)) increases deg(p(HGNC:TP53)).
- Binding of *PIAS1* to human *AR* DNA+ligand binding domains was *androgen* dependent in the yeast liquid beta-galactosidase assay. (PMID:10628744)
  a(CHEBI:androgen) directlyIncreases complex(p(HGNC:AR), p(HGNC:PIAS1)).

Example (a) shows a sentence with its target BEL statement. Two proteins <HGNC:AKAP11> and <HGNC:PPP1CC> are italicized while ‘decreases’ denotes the predicate of the relationship between the two proteins and *act()* (molecularActivity) is a biomedical function on the protein <HGNC:PPP1CC>. It states that the protein <AKAP11> decreases the molecular activity of the protein <PPP1CC>. Example (b) demonstrates an example where one sentence may correspond to multiple BEL statements and (c) is an example that complex function [*complex()*] which acts on two or more entities can be involved in a BEL statement.

Various approaches have been proposed to address the BEL task. They can be roughly grouped into rule-based, cross-task and within-task methods.

Ravikumar *et al*. (10, 11) tested a rule-based semantic parser that is capable of handling complex syntactic structures involving connectives, events and anaphora. They achieved the start-of-the-art performance in BioCreative V BEL Task, which demonstrates that domain-specific knowledge plays an important role in the task. However, the method has the issues of inflexibility and domain dependence. Cross-task methods convert intermediate structures predicted from other tasks into BEL statements. Choi *et al*. (12) extracted Genome Information Acquisition (GENIA) event structures using the Turku event extraction system (13) and then translated them into BEL statements. Lai *et al*. (14, 15) identified casual relations from the output of a biomedical semantic role labeler and classified entity functions with keywords appearing in the context of entities. Nevertheless, they did not make use of the original BEL training corpus, thus limiting their performance. Within-task methods directly use the BEL training corpus in one or the other way, hoping to improve the performance. Ali *et al*. (16) treated the BEL task as conventional binary RE and therefore can apply RE techniques directly. They used a Convolutional Neural Networks (CNN) model to extract the relationship between two biomedical entities. Other complex relations and biomedical functions are totally ignored, and, therefore, the performance is greatly diminished. Liu *et al*. (17) cast the BEL task as a hierarchical sequence-labeling problem. They constructed a training corpus from the original BEL training corpus using word alignment technique. However, due to the complexity of the task, training a model to directly extract BEL statements does not yield promising results.

In order to make full use of the BEL training corpus and include as many relations (including functions) as possible, we propose a method to extract BEL statements by combining RE with function detection (FD). Relations between two entities and biomedical functions related to these two individual entities are considered when generating a BEL statement in order to improve the overall performance. Two respective attention-based bidirectional long short-term memory networks (att-BiLSTM) models are used for RE and FD due to their excellent performance in the general domain (18). However, preliminary experiments show that simply merging the results from RE and FD did not yield performance improvement for BEL statement extraction. Therefore, a strategy of threshold filtering is applied to improve the precision of identified entity functions by discarding unreliable ones. Our contributions include the following:

- An att-BiLSTM model to detect entity function in order to incorporate them with entity relations into BEL statements.
- A strategy of threshold filtering to select entity functions with high reliability in order to improve the overall performance.
- We achieve the best F1 performance of 46.9% in stage 2 and 21.3% in stage 1 at statement level on the BioCreative V BEL task.

## Materials and methods

In this section, we first present the statistics on the corpus, then we systematically describe our approach for the BEL statement extraction task.

### Data set

The corpus provided by the organizer for the BioCreative V BEL task comprises the training, sample and test sets, where one sentence is annotated with one or more BEL statements. Table 1 reports the statistics on the sentence, BEL statements, entities, relations and functions in the BC-V BEL corpus as four parts from top to down as follows:

- The number of sentences and their associated BEL statements. Usually the latter is much greater than the former since there may be multiple BEL statements corresponding to one sentence.
- The number of four types of biomedical entities, gene/protein, chemical, disease and biological process. Among them, ~85% are gene/protein.
- The number of relations (‘increases’ and ‘decreases’), where ‘directlyIncreases’ and ‘directlyDecreases’ are mapped to ‘increases’ and ‘decreases’, respectively. Over 70% of the relations in the training set are ‘increases’. Notice that the total number of relations in the corpus is more than that of BEL statements. This is because nested relations in a BEL statement are decomposed into multiple binary relations.
- The number of major types of functions, among which over 65% are Activities. Main subtypes of Activities, Transformations and Modifications are also included in the parentheses. Usually the number of functions is less than that of entities involved in relations. This means that only a small number of entities in relations have functions.

**Table 1:** Statistics on the BC-V BEL task corpus

Statistics	Train	Sample	Test
Sentence	6353	190	105
BEL statement	11 066	295	202
Gene/Protein	14 108	333	238
Chemical	677	69	23
Disease	207	43	11
Biological process	1522	62	23
Total	16 514	507	295
*Increases*	8382	228	154
*Decreases*	3006	94	53
Total	11 388	322	207
Activities (*cat(), kin()*…)	4571	213	44
*Complex()*	659	26	16
Transformations (*deg()*…)	454	25	10
Modifications (*pmod()*…)	1212	24	9
Total	6896	282	79

From the above statistics on the training corpus, we can see that ~91% of relations are binary between two entities while only a small number of them contain nested relations. Focus on binary relations, therefore, will lose very few BEL statements with nested relations. Furthermore, among entities which appear in BEL statements, ~42% have a function with one entity as its argument, meaning that disregarding these functions would significantly hurt the overall performance (16). Therefore, in this work when we build BEL statements, we focus on the entities that have a binary relation and their unary functions.

## Methods

In our approach the BEL task is decomposed into two subtasks: entity RE and entity FD. First, binary relations between two entities are extracted and then entity functions involved in these relations are recognized via a new FD method. Finally, BEL statements can be formed by combining entity relations with their functions.

**Figure 1:**
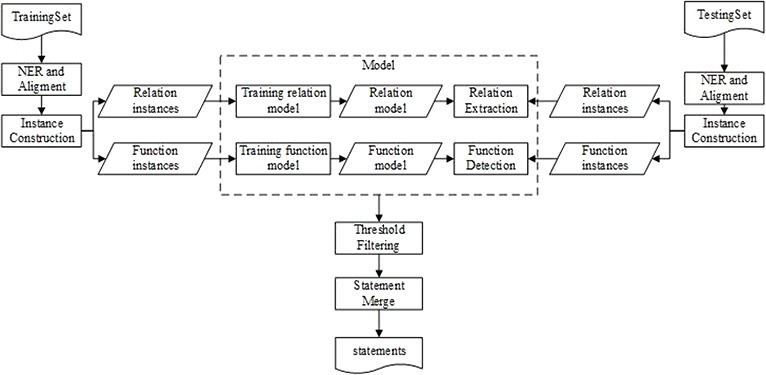
The system workflow diagram.

Figure 1 illustrates the workflow of our method that comprises five main components: name entity recognition and alignment (NERA), instance construction (IC), RE, FD, followed by BEL statement merging. The NERA module recognizes entities in a sentence and align them with the identifiers in BEL statements. The IC module constructs both RE and FD instances for training and testing, respectively. Then, two respective models for RE and FD are induced from the training instances. During testing, the RE and FD models are simultaneously applied to the testing instances to determine the relationship between two entities and their individual functions. Finally, based on the predictions of RE and FD, a BEL statement can be created for the pair of entities via BEL statement merging.

**Figure 2:**
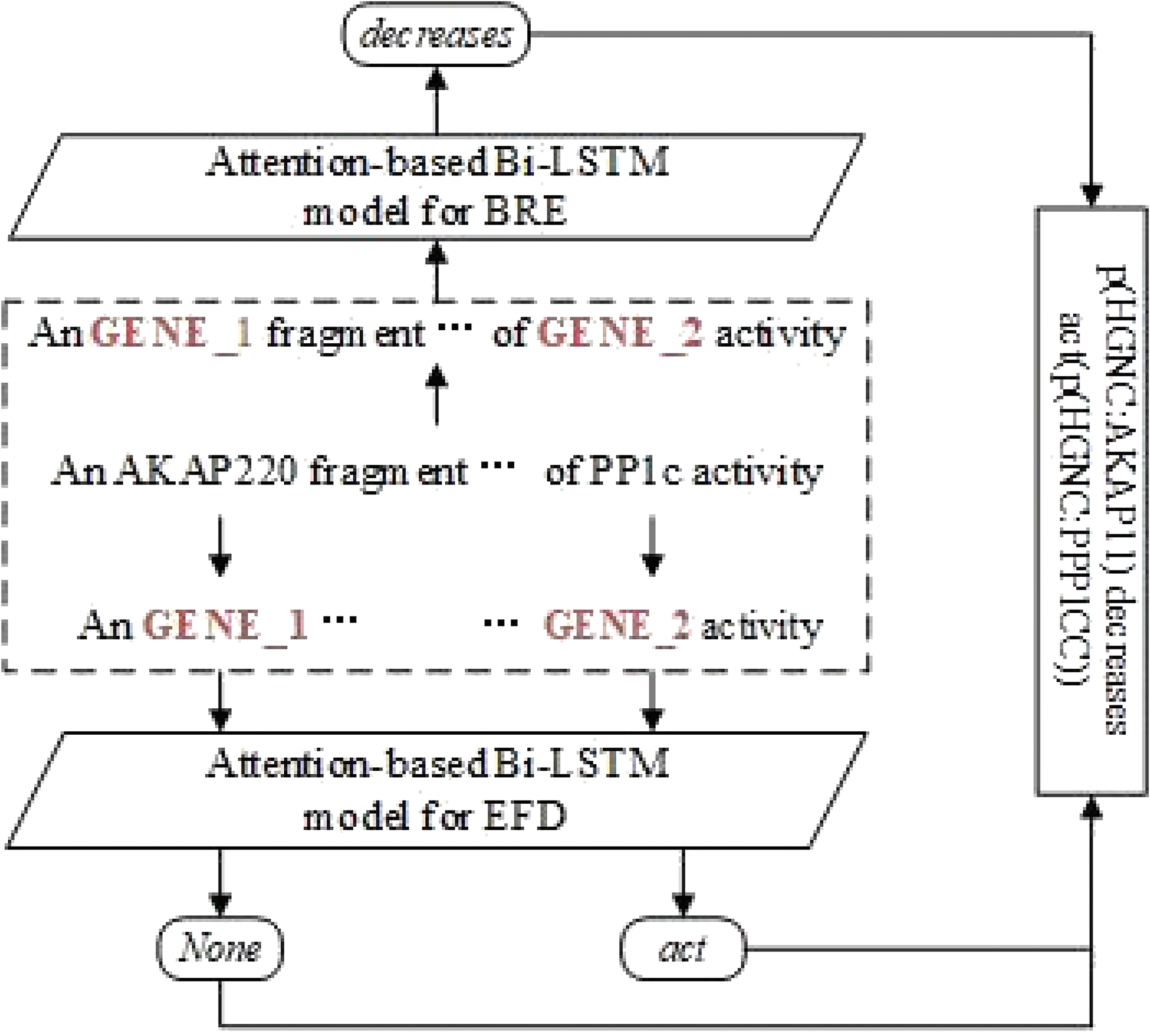
An example of the BEL statement extraction workflow.

Figure 2 shows an example of the BEL statement extraction workflow. The sentence in example (i) is the input. After the two entities are recognized and aligned in the sentence, the RE model is applied to extract the relationship between them. The FD model is applied to detect respective functions of two involved entities. Finally, the relationship and the functions are combined to form the output, i.e. the BEL statement ‘p(HGNC:AKAP11) decreases act(p(HGNC:PPP1CC))’.

### Name entity recognition and alignment

Since only the identifiers of entities, rather than their exact locations in a sentence, are given in the training corpus, the first step is to recognize biomedical entities in the sentence and align them to their identifiers in the BEL statement. We adopted three steps including NER, renormalization and dictionary search in order to maximize the entity recall.

`NER’. Three NER tools are used respectively to identify different biomedical entities, including GNormplus (19) for gene and protein recognition, tmChem (20) for chemical recognition and DNorm (21) for disease recognition. In addition, these tools also normalize recognized entities to the corresponding entity databases. GNormplus links genes and proteins to Entrez (22), tmChem links chemicals to Medical Subject Headings (MESH) (23) and CHEBI (24) and DNorm links diseases to MESH and OMIM (25). The normalized entities are finally aligned to their identifiers in the BEL statement.

`Renormalization’. Due to name variation, entity identifiers in the BEL statement, however, are not always the same as the ones recognized by the NER tools, so the second step is to renormalize and align the latter into the former. Protein identifiers are consistent across Entrez, HGNC and MGI, so no conversion is needed. Recognized chemical identifiers are converted to CHEBI ones in terms of their normalized names. Recognized disease identifiers are discarded if they are linked to OMIM since conversion from OMIM to MESH is currently infeasible.

`Dictionary search’. Although the three tools achieve the state-of-the-art performance in recognizing different biomedical entities, there are still a number of entities in the BEL statement unrecognized, particular for biological processes. Therefore, we finally performed a dictionary-based entity search for the remaining unaligned entities in the BEL statement. The dictionary consists of symbols and synonyms from five entity lists provided by the organizer, i.e. Mouse Genome Informatics (MGI), HUGO Gene Nomenclature Committee (HGNC), CHEBI, Medical Subject Headings from the Diseases (MESHD) and Gene Ontology names for Biological Process (GOBP), etc. The matching is based on edit distance and the continuous word sequence with minimal distance to the dictionary entries is recognized as the correct entity and aligned to the BEL statements.

For eliminating the variability of entity names and their lengths, we anonymize the entity mentions in sentences by replacing them with placeholders to indicate their types and numbers as GENE_1, GENE_2 as in Figure 2.

### Candidate instance construction

Prior to RE and FD, relation and function instances for both training and testing should be first constructed. Relation instances are generated from all entity mentions in a pairwise way. That is, if a sentence has *n* entities, it will produce *n*(*n*-1)/2 relation instances. Specifically, during training, if a relation candidate appears in the BEL statements, it is a positive instance with the corresponding relation type, otherwise regarded as a negative instance. In this way, we can generate the RE training set. At the same time, the FD training set is also generated from the BEL training set. For each entity in the BEL training set, if a function is associated with the entity, a positive function instance is generated, otherwise a negative instance is formed. If there are *m* positive relation instances in a sentence, 2*m* entity function instances will be produced. During testing, the relation and function instances are generated in a similar way except that an FD test instance is formed for each individual entity.

Using the above method, we generate a RE training set including 9149 positive, 4574 negative instances and an FD training set including 5226 positive instances and 9769 negative instances.

### Relation extraction

RE aims to extract the relationship between two entities, disregarding the functions around them, such as the relation type ‘decreases’ between the entity pair <p(HGNC:AKAP11)> and <p(HGNC:PPP1CC)> in the sentence without considering the function *act()*. The problem can be cast as a conventional RE problem, where an att-BiLSTM model is trained on the RE training set and then used to extract the relation on the RE test set because this kind of model has been demonstrated to perform excellently in RE in the general domain (18). The training instances (sentences and their relation labels) are fed into a learner to derive a classification model that is in turn used to predict the relation labels for the test instances. The RE model is elaborated in the Subsection **Models** simultaneously with the FD model because they share many similarities.

### Function detection

Entity FD is aimed to detect the functions of entities. For simplicity here we focus on the functions used to modify one entity, i.e. unary functions, excluding the *complex()* function involving two or more entities. As example (a) mentioned above, there are two entity function candidates, function *None* for <p(HGNC:AKAP11)> and function *act* for <p(HGNC:CASP1)>, respectively, in the BEL statement ‘p(HGNC:AKAP11) decreases act(p(HGNC:PPP1CC))’. There is no direct research on FD in the previous within-task methods, which is either regarded as a part of sequence labeling task (17) or totally discarded (16). Based on the observation that the function of an entity may depend on its context, we recast the subtask as a classification problem similar to RE except that there is only one entity involved. Therefore, an att-BiLSTM model is introduced to FD. The context within a window around an entity together with the function label is fed into the learner to induce the model which is in turn applied to predict the test instances. The FD model is also detailed in the section **Models**.

### BEL statement merging

After the identification of relations between two entities and their individual functions, it is straightforward to combine them into BEL statements. In Figure 2, for example, we first identify the relation ‘decreases’ between <GENE_1> and <GENE_2>, then detect the function *act* on the second protein, finally, we recover <GENE_1> and <GENE_2> to their normalized identifiers <p(HGNC:AKAP11)> and <p(HGNC:PPP1CC)>, finally, the relation ‘decreases’ and function *act* are combined into the corresponding BEL statement ‘p(HGNC:AKAP11) decreases act(p(HGNC:PPP1CC))’.

However, preliminary experiments showed that naive merging of entity functions into entity relations leads to overall performance degradation due to the relatively low precision of entity FD. Therefore, a strategy of threshold filtering is proposed to filter out the predicted functions with low reliability before merging in order to improve the overall performance. The idea behind the strategy is that unreliable functions hurt the accuracy of BEL statements when they are incorporated into entity relations.

### Models

**Figure 3:**
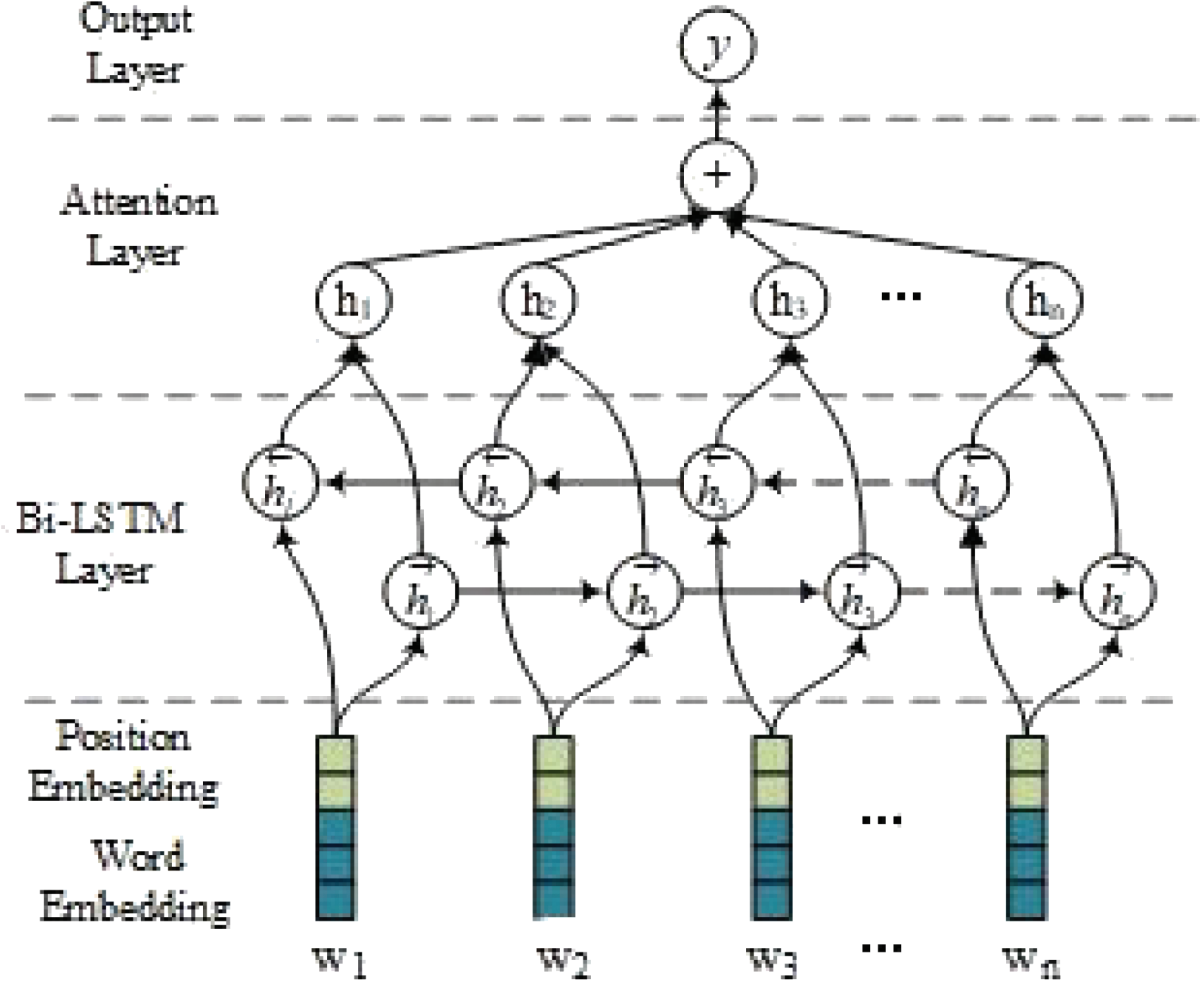
An att-BiLSTM model.

In this section, we describe in detail the att-BiLSTM model for both RE and FD. An overview of our model is illustrated in Figure 3, which includes the following four layers: embedding layer, Bi-LSTM layer, attention layer and output layer. The main difference between RE and FD lies in the input forms and the output labels.

The inputs of the networks for the entity RE and entity FD are slightly different. The input to RE is the tokenized words of the whole sentence while a window of 15 words around the entity is fed into the entity FD. In the embedding layer, an input word is encoded with the concatenation of the word vector and the position vector. Note that there are two position vectors corresponding to two entities for RE while there is only one position vector for FD. Then, the Bi-LSTM layer is followed to encode the sematic information of the input sequence. After that, the attention mechanism is applied to merge hidden vectors from each time step in the sequence into a sentence-level vector by multiplying them with their associated weights. Finally, a *softmax* transformation is used in the output layer to transform the sentence representation into a probability for a relation/function label. For entity FD, threshold filtering is further applied to filter out entity functions with low reliability.

### Input representation

Given an input sequence consisting of $\mathrm{\textit{n}}$ words $\mathrm{s}=\{{w}_1,{w}_2,\dots, {w}_n\}$ and two marked entities *e*_1_ and *e*_2_ or one marked entity *e*_1,_ we first convert each word into a real-valued vector. A word ${\mathrm{w}}_i$ is transformed into its word embedding ${{\mathrm{x}}^{\mathrm{w}}_{\mathrm{i}}}$ by looking up the word in the embedding matrix ${\mathrm{E}}^w\in {\mathbf{\mathbb{R}}}^{d_w\times \mid V\mid }$, where $\mathrm{\textit{V}}$ is the input vocabulary and ${\mathrm{d}}_{\mathrm{w}}$ is the word embedding dimension.

It is evident that words close to the target entities play a key role in determining the relation/function, so we also incorporate the word position embedding proposed by Zeng *et al*. (26). In the above sentence, the relative distances of ‘inhibitor’ to ‘AKAP220’ and ‘PP1c’ are 5 and −2, respectively. The relative distance is mapped to a vector with dimension ${\mathrm{d}}_{\mathrm{p}}$, which is a hyperparameter to be chosen. Let ${x}_{i,1}^p,{x}_{i,\,2}^p\in {\mathbf{\mathbb{R}}}^{d_p}$ denote the position vectors corresponding to the *i*-th word in the sentence for the first and second entities, respectively. The final embedding ${\mathrm{x}}_i$ for the *i*-th word can be obtained by concatenating the word embedding with the position vectors, i.e. for RE ${\mathrm{x}}_{\mathrm{i}}=\{{x}_i^w,{x}_{i,1}^p,{x}_{i,\,2}^p\}$, for entity FD, ${\mathrm{x}}_{\mathrm{i}}=\{{x}_i^w,{x}_i^p\}$. Accordingly, the input representation for the sequence can be represented $\mathrm{S}=[{x}_1,{x}_2,\dots, {x}_n]$.

### Bi-LSTM layer

The LSTM architecture consists of a set of recurrently connected cells, known as memory units. Each time step corresponding to each word is regarded as an LSTM memory unit, which is used to compute current hidden vector *h*_*t*_ based on the previous hidden vector *h*_*t*-1_ and the current input word embedding *x*_*t*_. Its operations can be defined as follows:

(1) \begin{equation*} {i}_t=\sigma \left({W}_{wi}\cdot {x}_t+{W}_{hi}\cdot {h}_{t-1}+{b}_i\right) \end{equation*}

(2) \begin{equation*} {\mathrm{f}}_t=\sigma \left({W}_{wf}\cdot {x}_t+{W}_{hf}\cdot {h}_{t-1}+{b}_f\right) \end{equation*}

(3) \begin{equation*} {\mathrm{g}}_t=\tanh \left({W}_{wc}\cdot {x}_t+{W}_{hc}\cdot {h}_{t-1}+{b}_c\right) \end{equation*}

(4) \begin{equation*} {\mathrm{c}}_t={i}_t\otimes {g}_t+{f}_t\otimes {c}_{t-1} \end{equation*}

(5) \begin{equation*} {\mathrm{o}}_t=\sigma \left({W}_{wo}\cdot {x}_t+{W}_{ho}\cdot {h}_{t-1}+{b}_o\right) \end{equation*}

(6) \begin{equation*} {\mathrm{h}}_t={o}_t\otimes \tanh \left({c}_t\right) \end{equation*}

where *i, f* and *o* are the input, forget and output gates, respectively, *b* is the bias terms, *c* is the cell memory and *W*_(…)_ are the training parameters. For each word *w*_*t*_, the forward LSTM layer will encode *w*_*t*_ by considering the contextual information from word *w*_1_ to *w*_*t*_, which is marked as $\overrightarrow{{\mathrm{h}}_{\mathrm{t}}}$. In a similar way, the backward LSTM layer will encode *w*_*t*_ based on the contextual information from *w*_*n*_ to *w*_*t*_, which is marked as $\overleftarrow{{\mathrm{h}}_{\mathrm{t}}}$. Finally, we use element-wise sum to combine $\overrightarrow{{\mathrm{h}}_{\mathrm{t}}}$ and $\overleftarrow{{\mathrm{h}}_{\mathrm{t}}}$ as the representation of the word’s encoding information, denoted as ${\mathrm{h}}_{\mathrm{t}}=\overrightarrow{{\mathrm{h}}_{\mathrm{t}}}\oplus \overleftarrow{{\mathrm{h}}_{\mathrm{t}}}$.

### Attention layer

It is obvious that not all words contribute equally to the representation of the sequence meaning. To illustrate this, we take the aforementioned sentence (a) as an example. It is intuitive that the importance of the word ‘inhibitor’ is much higher than other words when considering the semantic relation type of ‘decreases’. Therefore, we introduce the attention mechanism proposed by Zhou *et al*. (18) to automatically focus on the words that have decisive effects on relation classification or FD. The importance score ɛ_*i*_ of the *i*-th word in a sequence is given by:

(7) \begin{equation*} {\varepsilon}_{\mathrm{i}}={W}_a\cdot \tanh \left({\mathrm{h}}_i\right) \end{equation*}

Then, the normalized importance weight α_*i*_ for each word can be obtained through a *softmax* function shown as follows:

(8) \begin{equation*} {\alpha}_{\mathrm{i}}=\frac{\exp \left({\varepsilon}_{\mathrm{i}}\right)}{\sum_{k=1}^n\exp \left({\varepsilon}_k\right)} \end{equation*}

Where *h*_*i*_ is the *i*-th output vector the LSTM layer, and *W*_*a*_ is a weight vector to be learned during the training process. The dimension of both *h*_*i*_ and *W*_*a*_ is *d*_*w*_. Then the representation *r* of the sentence is formed by a weighted sum of all output vectors:

(9) \begin{equation*} r=\sum \limits_{i=1}^{\mathrm{n}}{h}_i{\alpha}_i \end{equation*}

### Output layer and threshold filtering

In the output layer, the sequence representation *r* is first non-linearly transformed to a vector ${h}^{\ast }$:

(10) \begin{equation*} {h}^{\ast }=\tanh (r) \end{equation*}

Then a *softmax* classifier is used to predict label 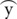 among a set of classes *y* from the vector ${h}^{\ast }$ as follows:

(11) \begin{equation*} \mathrm{p}\left(y|s\right)= soft\ \textit{max} \left({W}^{(s)}{h}^{\ast }+{b}^{(s)}\right) \end{equation*}

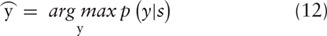


**Table 2:** Hyperparameters for both RE and FD models

Parameters	Value
Dimension of word embedding	200
Dimension of position embedding	64
LSTM units	600
Learning rate	0.001
Loss function	Cross-entropy
Regularization	L_2_
Regularization coefficient	0.0001
Optimizer	Adam

Here a difference exists between RE and FD. For the former, we just take 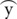 as the output relation label. For the latter, however, we introduce a threshold τ to filter out unreliable entity functions in order to improve the precision though at the expense of the recall. If the probability of 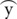 is lower than τ, we relabel the instance as a negative one. That is


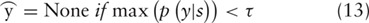


The idea behind the threshold filtering is that if the precision of FD is too low, it will significantly degrade the performance of BEL statements as will be demonstrated in Table 5.

### Training

To learn the parameters of the networks, we adopt the following loss function for training both RE and FD models:

(14) \begin{equation*} \boldsymbol{J}\left(\theta \right)\in -\frac{1}{m}\sum \limits_{i=1}^m\log p\left({y}_i|{\mathrm{s}}_i,\theta \right)+\lambda {\left\Vert \theta \right\Vert}^2 \end{equation*}

where $p({y}_i|{\mathrm{s}}_i,\theta)$ is the confidence score of the gold label *y_i_* of the training relation/function instance, *λ* is the regularization coefficient and *θ* is the set of parameters.

### Experimentation

In this section, we first present the hyperparameters of our models, then we describe the evaluation, finally, we systematically evaluate the performance of our approach on the corpus.

### Hyperparameter setting

We adopt the same set of parameters as listed in Table 2 for both RE and FD models due to their similar structure. Particularly, word embeddings are randomly initialized and further automatically adjusted during the training process, since preliminary experiments didn’t show any improvements for pre-trained word embedding.

### Evaluation metrics

The performance is measured in terms of standard P/R/F1; however, due to the complexity of BEL statement extraction, different levels of scores are also calculated in order to evaluate the performance at different extraction levels, i.e. Term (T), Function-Secondary (FS), Function (Fun) Relation-Secondary (RS), Relation (Rel) and Statement (Stat). In particular, evaluation scheme does not discern between direct and indirect relation types*,* which means that ‘increases’ and ‘directlyIncreases’ are treated as equal, so are ‘decreases’ and ‘directlyDecreases’, and function evaluation is simplified by mapping activity functions, such as *kin(), tscript() and cat(),* to the more general *act()* function (27). Among them the statement one is the overall performance that we are concerned with. The evaluations are done on the BC-V test set with gold entities (stage 2) unless it is specified that entities are automatically recognized (stage 1). For more information about the BC-V BEL task and its evaluation, kindly refer to Rinaldi *et al*. (27) and Fluck *et al*. (28).

### Experimental results

#### Cross-validation performance of RE and FD on the BC-V training set

We evaluate the cross-validation performance of our models on the RE and FD, respectively, where we apply a 10-fold cross-validation to the RE and FD training sets. The average results across 10-folds are reported in Table 3 where RE and FD denote the overall performance for RE and FD, respectively. The best performance scores in each column for individual relations and main functions are displayed in bold typeface. The values in the parentheses beside the F1-scores are their standard deviations across 10-folds.

**Table 3:** 10-fold cross-validation performance of RE and FD on the BC-V training set

Relation/function types	#	P(%)	R(%)	F1(%)
RE	9176	61.7	60.8	61.3(±1.4)
*Increases*	6701	**65.1**	**73.4**	**69.2**(±1.4)
*Decreases*	2475	54.2	40.0	46.0(±2.4)
FD	5226	53.9	54.0	53.9(±2.5)
*act()*	4163	52.3	**59.8**	**56.0(±4.1)**
*deg()*	103	58.8	16.9	26.3(±16.)
*pmod()*	698	**59.5**	32.8	42.3(±5.8)
*sec()*	226	51.7	23.1	31.9(±9.2)

Table 3 shows that causal RE and FD in biomedical domain are two challenging subtasks with 61.3 and 53.9% of overall F1-measures, respectively. It also shows that

- The performance of FD is lower than that of RE. This is mainly because the classes of entity relations (2) is less than that of entity functions (4) and the RE training set is much bigger than the FD one.
- For the subtask of RE, the performance of the type ‘decreases’, especially its recall, is drastically lower than that of ‘increases’. Obviously, it is due to the great number of training instances for ‘increases’.
- For the subtask of FD, the performance of *act()*, particularly its recall, is much higher than those of other functions. However, the precision of *pmod()* is the highest among all functions, probably because the expressions containing ‘phosphorylation’ usually denote the *pmod()* function.

#### Performance on the BC-V test set with/without functions

We evaluate our RE and FD models, which were induced from the whole RE and FD training sets respectively, on the BC-V test set with gold entities (stage 2). Due to variations for multiple runs of the same model trained on the same data set on the TensorFlow platform, we average the results over five runs. The same setting will be used in the following experiments unless specified otherwise. The upper part of the Table 4 shows the performance at various levels with naïve merging of relations and functions while the lower part shows the statement performance without/with functions, i.e. only relations and naive merging, respectively. Note that the function performance is only related to naïve merging while Term/Relation performance remains constant. From Table 4 we can see that

- The performance at T level is extremely high with around 95% or above for P/R/F1. This is because in stage 2 all the entities participating in BEL statements are given, and the high performance at RS level indicates that nearly all the relations are recognized in a loose sense, leading to the inclusion in the final BEL statements of all the entities involved in these relations.
- The performance at RS level is also surprisingly high with ~96% of F1. On the one hand, due to its loose criteria, RS level only evaluates whether any two of three arguments in a relation instance (i.e. subject, predicate and object) match rather than all its three arguments. On the other hand, our model is trained on the data set and applied to the test set where each gold entity should be involved in at least one relation, and thus RE in this scenario is relatively easier than in the general setting where a large number of negative instances dominate both training and test sets.
- There is a dramatic decline in performance from RS level to Rel level due to the latter’s strict evaluation criteria, i.e. all three arguments in a relation, including relation types and argument order, are evaluated. Therefore, errors in both relation types and argument order contribute to the performance decline.
- Compared with the merging strategy of only using RE, the F1-measure of naive merging at Stat level decreases 3 units (from 44.9 to 41.7%) when entity functions are incorporated into the statements. This is contrary to our intuition that entity functions would enhance the statement performance if they are detected correctly. We also notice that the function performance is significantly lower than that of cross-validation in Table 3 by ~20 units. This is mainly due to the errors caused in the predicted relations. After careful examination, we found that due to the low precision of FD (31.7%), more than half of predicted functions are wrong, leading to the corresponding incorrect BEL statements, otherwise some of these statements would be correct if no entity function is introduced.

**Table 4:** Performance in stage 2 on the BC-V test data with/without considering functions

Evaluation levels	P(%)	R(%)	F1(%)
Term	99.3	95.2	97.2(±0.7)
FS	**43.3**	**45.2**	**44.3(±2.3)**
Function	**31.7**	**36.7**	**34.0(±2.9)**
RS	98.8	94.4	96.5(±0.7)
Relation	66.2	65.4	65.8(±0.8)
Statement(RE)	45.1	44.8	44.9(±1.0)
Statement(Merging)	**42.5**	**41.2**	**41.7(±1.6)**

**Figure 4:**
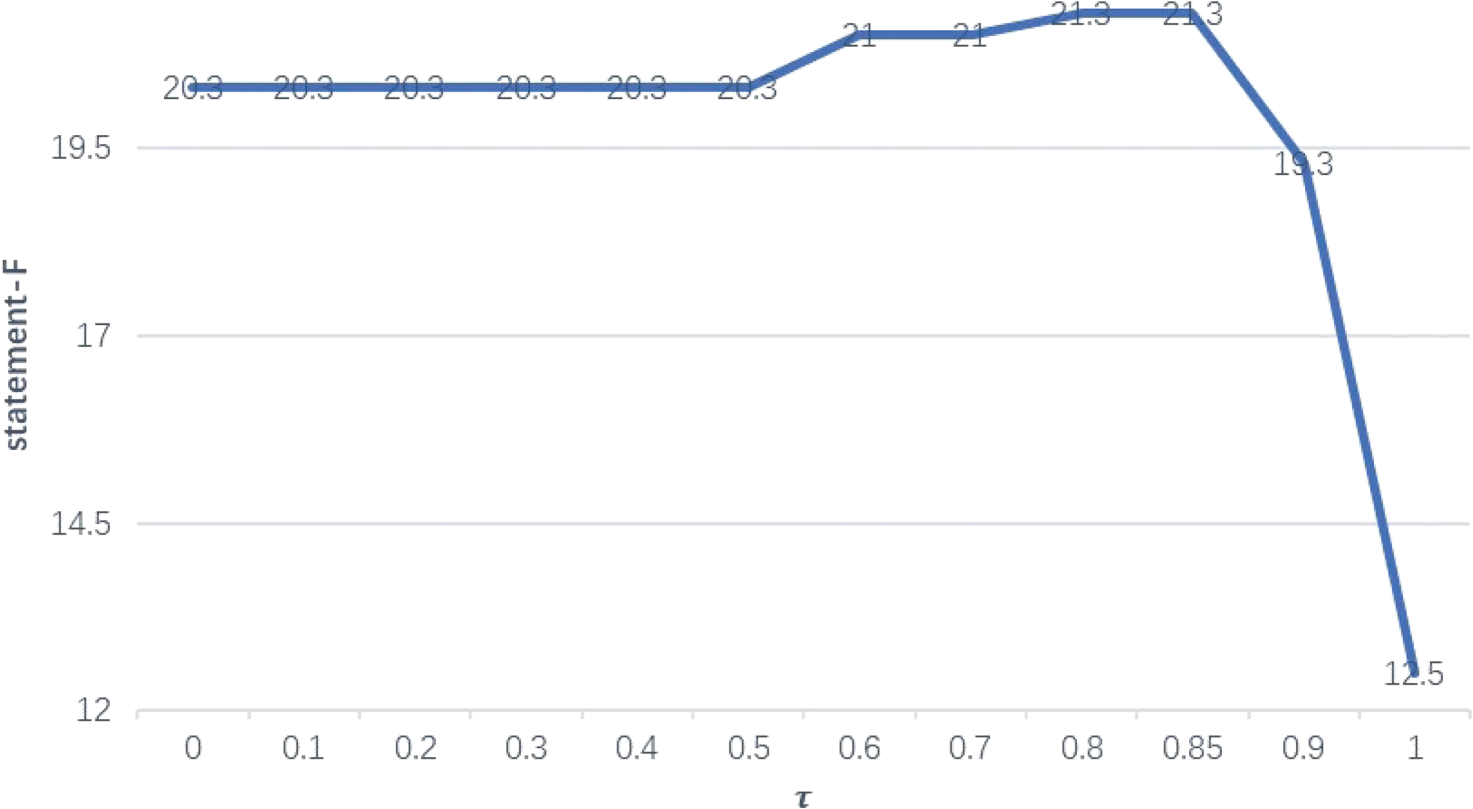
The performance of F1 with different thresholds on the BC-V sample set with gold entities.

#### Impact of threshold filtering on the BC-V sample set

In order to select the optimal threshold τ, we evaluate its impact on the statement performance on the BC-V sample set. Figure 4 illustrates how the F1-measures (on the vertical axis) on the sample set with gold entities vary with different thresholds (on the horizontal axis) from 0 to 1.

We can see in Figure 4 that when τ equals 0.8 or 0.85, the statement performance reaches the highest level, which results in an increase of 1.0 in F1-measure compared with when τ is between 0 and 0.5. Therefore, τ is set to 0.8 in the following experiments.

Two threshold values of 0 and 1 need to be particularly noted, where 0 implies naive merging of relations and functions and 1 means that only relations are considered. The significant decrease for τ=1 is due to the fact that, different from the test set, most (~70%) BEL statements on the sample set entail entity functions, and therefore omission of entity functions will significantly degrade the statement performance.

#### Performance on the BC-V test set using statement merging with threshold filtering

Table 5 reports the performance at different levels on the BC-V test set when τ is set to 0.8 for threshold filtering. Likewise, the performance scores different from those in Table 4 are displayed in boldface, it shows that

- With threshold filtering, we achieve the best statement F1-measure of 46.9% with the increases in both precision and recall. This justifies the strategy of threshold filtering due to the high reliability of functions as shown by the high precision scores at both function and function-secondary levels.
- Nevertheless, the recall scores at both Fun and FS levels are lower than those in Table 4. The reason is obviously due to threshold filtering that favors precision at the expense of recall degradation.

**Table 5:** Performance in stage 2 on the BC-V test set using relation merging with threshold filtering

Evaluation levels	P(%)	R(%)	F(%)
Term	99.3	95.2	97.2(±0.7)
FS	**71.6**	**23.1**	**34.8(±2.7)**
Function	**57.2**	**17.4**	**26.6(±1.6)**
RS	98.8	94.4	96.5(±0.7)
Relation	66.2	65.4	65.8(±0.8)
Statement	**47.5**	**46.3**	**46.9(±1.6)**

#### Performance in stage 1 on the BC-V test set using different merging strategies

In order to investigate whether threshold filtering can work when gold entities are not given, we test our models on the BC-V test set with automatically recognized entities and report in Table 6 the performance in stage 1. The top half of the table shows the performance at various levels using threshold filtering for merging statements (Actually, only performance of FS and Fun is related to merging strategies.) while the bottom part shows the statement performance using different merging strategies, i.e. only relations, naive merging and merging with threshold filtering, etc. Note that the same models as in Table 5 are used.

**Table 6:** Comparison of performance in stage 1 on the BC-V test set using different merging strategies

Evaluation levels	P(%)	R(%)	F(%)
Term	56.3	63.6	58.6(±0.9)
FS	66.7	23.1	34.3(±1.4)
Function	36.8	11.7	17.7(±2.1)
RS	57.6	67.8	62.3(±1.7)
Relation	27.7	36.6	31.6(±1.8)
Statement(RE)	16.0	23.3	19.0(±1.2)
Statement(Merging)	14.2	20.7	16.8(±3.2)
Statement(Filtering)	**18.7**	**24.8**	**21.3(±1.8)**

Compared with the performance in stage 2 in Tables 4 and 5 etc., a significant decrease in Table 6 occurs at all levels except FS. This should, at first sight, be caused by entities mistakenly recognized, but may also result from the fact that the RE model was trained on the biased training set where positive instances greatly outnumber negative ones decreases significantly while there are much more negative instances in the test set in stage 1.

Nevertheless, the statement performance in stage 1 shows a similar trend to the performance in stage 2 regarding different merging strategies. When naive merging is adopted, the statement performance actually decreases compared with using only relations, and merging with threshold filtering in stage 1 can also boost the statement performance probably due to the same reason as in stage 2.

#### Comparison with other systems

Table 7 compares the performance of our method on the BC-V BEL test set with other systems in stage 1 (the upper half) and stage 2 (the lower half). The other systems on the BC-V task are based on rule (10), event (12) and Semantic Role Labeling (SRL) (14). The highest performance in each column is displayed in boldface. (We select the best performance of other systems from all possible runs.)

**Table 7:** Performance comparison with other systems on the BC-V test set in stages 1 and 2

Systems	T	FS	Fun	RS	Rel	Stat
Rule (10)	**62.9**	**55.4**	42.6	**73.3**	**49.2**	39.2
Event (12)	34.0	10.0	8.6	25.1	41.4	20.2
SRL (14)	45.0	9.5	2.7	56.7	26.4	19.7
Ours	58.6	34.3	17.7	62.3	31.6	**21.3**
Rule (10)	82.4	**56.5**	**30.0**	82.4	65.1	25.6
Event (12)	54.3	26.1	20.8	61.5	43.7	35.2
SRL (14)	55.2	-	-	63.5	44.6	33.1
Ours	**97.2**	34.8	26.6	**96.5**	**65.8**	**46.9**

We can see in Table 7 that in stage 2, our system achieves the best performance at 4 of 6 evaluation levels except functions. At stat level, we achieve the F1-measure of 46.9%, significantly outperforming other systems by more than 10 units. In stage 1, our system still achieves competitive F1-measure, though in a lesser degree. This demonstrates that attention-based neural networks together with threshold filtering are promising for BEL statement extraction.

## Discussion

To understand why the task is challenging, we closely examined the errors and grouped them in terms of different stages.

- `Misaligned entity mentions’. The first step of our approach is to align entity identifiers in a BEL statement to entity mentions in the sentence. However, an entity identifier may be aligned to an erroneous mention in large part due to the dictionary search based on edit distance, particularly for biological processes. For example, the BEL statement ‘tscript(p(HGNC:JUN)) increases bp(GOBP:“wound healing”)’ corresponds to the sentence ‘These results demonstrate that activing B promotes epithelial wound closure in vivo through the RhoA-Rock-JNK-cJun signaling pathway’ (PMID: 21949871). Based on the edit distance between continuous words, the entity <HGNC:*JUN*> is mistakenly aligned to ‘wound’ and entity <GOBP:“*wound healing*” > is misaligned to ‘signaling’.
- `Long-distance dependence’. One error source for RE is that the relationship between two entities is determined by the long-distance dependence in the sentence, which is still very difficult to be captured by an att-BiLSTM model. For instance, the BEL statement ‘p(MGI:Egf) increases r(MGI:Tkt)’ corresponds to the sentence ‘In addition, TKT mRNA levels were elevated fivefold in the corneas of 28-day-old mice raised in darkness and injected with EGF compared to uninjected mice also deprived of light’ (PMID: 11095059). The long-distance dependence between ‘TKT’ and ‘EGF’ determines the relationship ‘increases’.
- `Lack of domain knowledge’. A large part of entity functions can only be inferred from domain knowledge other than the sentence. For example, the *kin()* function denotes that an entity acts as a kinase, in some cases, however, the sentence doesn’t express the function at all, as in ‘Mutant src(−/−) mice have osteopetrosis resulting from defective osteoclasts (increased apoptosis).’ (PMID: 11157779) with its corresponding BEL statement ‘kin(p(MGI:Src)) decreases path(MESHD: Osteopetrosis)’. The *kin()* function of the protein <MGI:Src> can only be inferred from its description ‘neuronal proto-oncogene tyrosine-protein kinase Src’ in the MGI database, indicating that it is a kinase.
- `Cascaded errors’. An unavoidable disadvantage of a pipelined system like ours is that errors from the previous step can be propagated and further amplified to the next one, leading to significant errors accumulated in the system. The misaligned entity mentions, long-distance dependence and the lack of domain knowledge all contribute the low performance for the whole system.

## Conclusion

In this work, we tackle the BEL statement extraction task as a combination of RE and FD. We adopt the state-of-the-art models (att-BiLSTM networks) to extract entity relation as well as detect their individual functions, followed by the incorporation of entity relations and functions to form the BEL statements. In order to boost the overall performance, we also introduce the strategy of threshold filtering to select the highly reliable functions before constructing BEL statements. Experimental results show that our method achieves the best performance on the BC-V BEL task.

The limitation in our work is that we do not tackle complex functions and nested relations that still account for a non-negligible number of relations. We will deal with these issues in the future work. We also intend to jointly train entity RE and FD in order to further improve the overall performance.

## Notes

**Database URLs**: https://wiki.openbel.org/display/BIOC/BioCreative+BEL+Task+Challenges
